# The genome of the live-bearing fish *Heterandria formosa* implicates a role of conserved vertebrate genes in the evolution of placental fish

**DOI:** 10.1186/s12862-019-1484-2

**Published:** 2019-07-26

**Authors:** Henri van Kruistum, Joost van den Heuvel, Joseph Travis, Ken Kraaijeveld, Bas J. Zwaan, Martien A. M. Groenen, Hendrik-Jan Megens, Bart J. A. Pollux

**Affiliations:** 1 Animal Breeding and Genomics Group, Wageningen University, Wageningen, The Netherlands; 2 Experimental Zoology Group, Wageningen University, Wageningen, The Netherlands; 3 Plant Sciences Group, Laboratory of Genetics, Wageningen University, Wageningen, The Netherlands; 4 Department of Biological Science, Florida State University, Tallahassee, USA; 5 Institute for Biodiversity and Ecosystem Dynamics, University of Amsterdam, Amsterdam, The Netherlands; 6 Leiden Genome Technology Center Department of Human Genetics, Leiden University Medical Center, Leiden, The Netherlands

**Keywords:** *Heterandria formosa*, Poeciliidae, Placenta, Matrotrophy, Positive selection, Gene duplication, Molecular evolution, Whole genome sequencing

## Abstract

**Background:**

The evolution of complex organs is thought to occur via a stepwise process, each subsequent step increasing the organ’s complexity by a tiny amount. This evolutionary process can be studied by comparing closely related species that vary in the presence or absence of their organs. This is the case for the placenta in the live-bearing fish family Poeciliidae, as members of this family vary markedly in their ability to supply nutrients to their offspring via a placenta. Here, we investigate the genomic basis underlying this phenotypic variation in *Heterandria formosa*, a poeciliid fish with a highly complex placenta. We compare this genome to three published reference genomes of non-placental poeciliid fish to gain insight in which genes may have played a role in the evolution of the placenta in the Poeciliidae.

**Results:**

We sequenced the genome of *H. formosa*, providing the first whole genome sequence for a placental poeciliid. We looked for signatures of adaptive evolution by comparing its gene sequences to those of three non-placental live-bearing relatives. Using comparative evolutionary analyses, we found 17 genes that were positively selected exclusively in *H. formosa*, as well as five gene duplications exclusive to *H. formosa*. Eight of the genes evolving under positive selection in *H. formosa* have a placental function in mammals, most notably endometrial tissue remodelling or endometrial cell proliferation.

**Conclusions:**

Our results show that a substantial portion of positively selected genes have a function that correlates well with the morphological changes that form the placenta of *H. formosa*, compared to the corresponding tissue in non-placental poeciliids. These functions are mainly endometrial tissue remodelling and endometrial cell proliferation. Therefore, we hypothesize that natural selection acting on genes involved in these functions plays a key role in the evolution of the placenta in *H. formosa*.

**Electronic supplementary material:**

The online version of this article (10.1186/s12862-019-1484-2) contains supplementary material, which is available to authorized users.

## Background

Explaining the evolution of complex organs, consisting of multiple interacting parts, is one of the greatest challenges in evolution. Charles Darwin was the first to propose an explanation for this phenomenon; in his seminal work on natural selection, he hypothesized that complex organs were not complex at first, but gradually evolved into what we observe today [1]. However, finding examples of this stepwise process poses a challenge, mainly because of two reasons. First, species possessing an organ of intermediate complexity have often gone extinct, leaving the present-day observer with only the end-result of a long series of potentially minute evolutionary steps. Second, when differences in organ complexity between species exist, these species are often separated by a large phylogenetic distance, sharing only a very remote common ancestor. For instance, intermediate stages of complexity can be found in the mollusc eye [2, 3]. However, the different types of mollusc eyes are found in distantly related taxa, which diverged about half a billion years ago. This makes a comparative analysis on a genomic level not straightforward. To truly understand how molecular pathways are altered during evolution to give rise to complex organs, a model system is required that has recently evolved a complex organ with the ancestral and intermediate states still extant in closely related species. Ideally, such a complex organ should have originated multiple times, e.g. due to convergent evolution resulting from similar evolutionary pressure. Such a model system can be found in the development of the placenta in the livebearing fish family Poeciliidae [4].

The placenta is an organ that facilitates nutrient exchange between mother and offspring. It is present in all major vertebrate lineages, although its anatomical details differ between taxa [5, 6]. Numerous genes involved in placental development have been identified, making the placenta a prime example of complexity [7–9]. Most research on the placenta has been performed in eutherian mammals. Eutherian mammals, however, are limited in their suitability to study the evolution of the placenta, because all contemporary placental mammals (i) inherited their placenta from a single common ancestor that lived > 160 million years ago, and (ii) all have complex placentas and have no close living relatives that lack placentas. By contrast, the placenta has been estimated to evolve independently nine times in amphibians, and 12 times in ray-finned fish [5].

There are three reasons to focus on placental evolution of the live-bearing fish family Poeciliidae. First, the placenta has evolved independently at least eight times in the Poeciliidae [10]. This makes it possible to compare different instances of placental evolution within closely related species. Second, intermediate stages of placental complexity exist within this family. In fact, placental complexity in the Poeciliidae seems to vary continuously amongst species, rather than species either having a placenta or not [4]. Third, all of this variation is present among relatively closely related species. This allows us to more easily compare the genomes of these species. A genomic comparison between species varying in placental complexity may unveil the genomic basis underlying this difference in complexity.

The degree of maternal provisioning in the family Poeciliidae has been quantified in the Matrotrophy Index (MI), which is the estimated dry mass of offspring at birth divided by the dry mass of the egg at fertilization [11]. Poeciliid fish have a MI ranging from 0.6 for non-placental (lecithotrophic) species to more than 100 for species with a highly complex placenta (matrotrophic), with species exhibiting intermediate values also being present [4]. The MI can act as a proxy for placental complexity, because species with a high MI have a more complex placenta compared to species with a low or intermediate MI [12–15]. The main differences lie in the structure of the maternal follicular epithelium. The unspecialized follicular wall of lecithotrophic (non-placental, MI < 1) species is very thin and plays no role in maternal provisioning [15, 16]. In matrotrophic (placental) species the follicular epithelium is much thicker, more extensively folded and features specialized adaptations that facilitate maternal-to-embryo nutrient transfer, such as a high vascularization, a high density of microvilli, and the presence of specialized cytoplasmic organelles [12, 14]. Given the co-occurrence of these structural tissue features with a high MI, it is likely that these adaptations facilitate extensive matrotrophy.

Early studies on natural selection at the molecular level in the family Poeciliidae have compared genes of one or more poeciliid species to genes of other more distantly-related teleosts [17–19], or the analysis was limited to one or only a few genes known to be involved in placenta development in mammals [18, 20]. Exhaustively identifying genes responsible for placentation is impossible in such approaches, because large differences in placental complexity exist *within* the family Poeciliidae. In the present study, therefore, natural selection is investigated between more closely related species, focusing on the genomic differences between lecithotrophic and matrotrophic species within the family Poeciliidae.

Here, we investigate the genomic basis of placental complexity by exploring the genome of a highly matrotrophic poeciliid: the least killifish, *Heterandria formosa*. This species has a MI of around 35, and morphological analysis has shown that it has a highly complex placenta [14]. Specifically, we aim to, (1) sequence the genome of *H. formosa*, providing the first whole genome sequence of a matrotrophic poeciliid, and (2) compare this genome to published reference genomes of three related lecithotrophic species: the Trinidadian guppy (*Poecilia reticulata*) [21], the Amazon molly (*Poecilia formosa*) [17], and the Platyfish (*Xiphophorus maculatus*) [18]. These latter three species are lecithotrophic (MI < 1), and lack a placenta. Such large difference in placentation in closely related species may suggest the involvement of natural selection, which should be visible in associated signatures of selection in the genome. Comparing genes evolving under positive selection to their orthologs in three non-placental species allows prioritization of genes related to placentation; genes showing evidence of positive selection in *H. formosa*, but not in any of its lecithotrophic relatives are likely enriched for involvement in placentation. Additionally, we identified genes that have likely been duplicated in the genome of *H. formosa*, using a combination of breakpoint and read-depth based methods. Gene duplications are known to be an important driving force of adaptive evolution, so it is plausible that an increased placental complexity is associated with distinct gene duplications [22]. Through these methods we identify a number of genes that have likely contributed to phenotypic variation in, and evolution of, placentation in the family Poeciliidae.

## Results

### Whole genome sequencing of *Heterandria formosa*

We sequenced the genome of *H. formosa* to an average coverage of 40X, yielding 90 Gb data containing 182 million 150 bp paired-end reads. The genome was assembled using SPAdes assembler [23], resulting in a draft assembly with a size of 722 Mb. *H. formosa* genome size estimation based on k-mer analysis showed an estimated genome size of 670 Mb, which is slightly lower than the assembly size. This is possibly a result of the relatively high heterozygosity of the sample leading to redundant contigs, as the sequenced individual was not from an inbred population. To reduce this redundancy, redundans [24] was run on the assembly to remove heterozygous contigs, and rescaffold the assembly based on paired-read information. This reduced the assembly size to 608 Mb, which is slightly lower than the estimated genome size, and also lower than other poeciliid genome assemblies [17, 18, 21]. Additionally, scaffold N50 increased from 11 Kb to 26.5 Kb by the rescaffolding procedure. The lower assembly size compared to the estimated genome size can be explained by the fact that this assembly was based on short reads, and some repetitive sequences will likely be collapsed in the assembly, leading to a somewhat smaller assembly size. Summary statistics of this genome assembly are listed in Table 1.

**Table 1:** Summary statistics for the *H. formosa* genome assembly

Assembly size	608 Mb
Contig N50	6108 bp
Largest contig	77373 bp
Scaffold N50	26563 bp
Largest scaffold	226934 bp
GC content	38.59%
Heterozygosity	1 in 203 sites

**Fig. 1:**
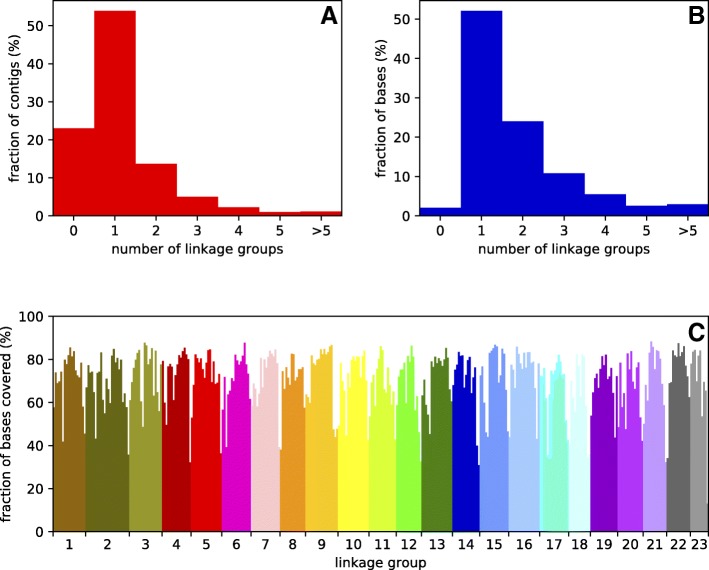
**a** Fraction of *H. formosa* assembly contigs aligning to a certain number of linkage groups of the *P. reticulata* genome assembly. **b** Fraction of bases in *H. formosa* contigs that align to a certain number of linkage groups of the *P. reticulata* genome assembly. **c** Percentage *of P. reticulata* bases covered by the 1:1 *H. formosa*: *P.reticulata* alignment, 2 Mb bins

The genome of *H. formosa* was aligned to the reference genome of *P. reticulata* using LAST [25] . The majority of the scaffolds of the *H. formosa* assembly aligned to one linkage group in *P. reticulata* (Fig. 1b), suggesting extensive synteny between the two species. For some smaller contigs, no match to *P. reticulata* linkage groups was found (Fig. 1a). All *P. reticulata* linkage groups were covered roughly equally by the *H. formosa* contigs, covering around 80% of the bases in *P. reticulata* (Fig. 1c). This means that around 20% of the *P. reticulata* bases were not covered by any *H. formosa* sequence, which may be because the *H. formosa* assembly is smaller than the *P. reticulata* assembly, or because there is high sequence divergence in these regions.

The coverage drops at the edges of the linkage groups, likely reflecting the underrepresentation of repetitive sequences in the *H. formosa* assembly due to it being assembled from paired-end reads only. A portion of the *H. formosa* contigs (23% base fraction) was split in the alignment to two *P. reticulata* linkage groups (Fig. 1b). For a minority (10%) of these contigs, alignment length was longer than 1000 bp for both linkage groups to which the contig aligned. This observation suggests that some genomic rearrangements may have occurred. For contigs aligning to three or more linkage groups, alignments were generally very short for all but one linkage group, indicating that this is most likely a result either of contigs aligning to ambiguous regions in the genome, or assembly errors.

### Positive selection

We identified 8,056 1:1:1 gene orthologs between *P. reticulata*, *P. formosa* and *X. maculatus* using ProteinOrtho [26]. From these genes, we retrieved the complete coding sequences of 6,774 genes in *H. formosa* through the whole genome alignment with *P. reticulata*. Using the codeml program of the PAML package [27], we tested these genes for both positive selection across all investigated poeciliid species, and positive selection in *H. formosa*. At 10% FDR, we found 104 genes to be positively selected across the whole phylogeny and 29 genes to be positively selected in *H. formosa*. Eleven genes were significant for both tests, leaving 18 genes exclusively positively selected in the *H. formosa* lineage (Table 2). In one case, a stop-gained mutation was observed inside the first exon, so this protein was left out of the final results.

**Table 2:** positively selected genes in *H. formosa* (10% FDR)

Gene symbol	gene name	*p*-value
*Pla2g2a*	Phospholipase A2 Group IIA	3.32E-07
*Timp4*	Tissue Inhibitor Of Metalloproteinases 4	2.92E-06
*Rbl1*	Retinoblastoma-Like 1	1.63E-05
*Cldnd*	Claudin d	2.28E-05
*Tmem230*	Transmembrane Protein 230	3.34E-05
*Kiaa1324/Eig121*	Estrogen Induced Gene 121	3.43E-05
*Pnkd*	Paroxysmal Nonkinesigenic Dyskinesia	6.69E-05
*Mmp15*	Matrix metalloproteinase 15	2.50E-04
*Gpr34*	G Protein-Coupled Receptor 34	2.55E-04
*Btbd7*	BTB Domain Containing 7	2.68E-04
*Glp1*	Glucagon-like peptide 1	2.84E-04
*Cldn4*	Claudin 4	3.04E-04
*Slc35d3*	Solute Carrier 35 Member d3	3.27E-04
*Pcdh10*	Protocadherin-10	4.05E-04
*Loc103465290*	Uncharacterized protein	4.51E-04
*Allc*	Allantoicase	4.58E-04
*Slc20a1*	Solute Carrier Family 20 Member a1	5.60E-04

A substantial number of these genes have placental functions in mammals. First, *Pla2g2a* was isolated from human placenta [28], and evidence found in horse points to a function in placental steroid metabolism [29]. However, activity of this protein is not limited to placenta, and has been linked to the immune system as well [30]. Second, a matrix metalloproteinase and a matrix metalloproteinase inhibitor (*Mmp15* and *Timp4*) were both positively selected in *H. formosa*. Both proteins are involved in endometrial tissue remodelling and placental labyrinth formation [31, 32]. Third, *Rbl1* and *Kiaa1324* gene expression has been linked to endometrial cell proliferation [33, 34]. Fourth, two claudin proteins (*Cldnd* and *Cldn4*) were found to be positively selected in this analysis. Claudins are cell-cell adhesion proteins known to be essential in placental tight junctions, regulating ion transport [35, 36]. Interestingly, claudins are also involved in tissue remodelling by interacting with matrix metalloproteinases [37, 38]. Finally, *Btbd7* is involved in tissue remodelling of embryonic epithelial cells by interacting with cell-cell adhesion proteins [39], and is associated with preeclampsia in humans [40]. We searched for expression of these genes in the human protein atlas [41] and the tissue-specific transcriptome of the closely related *Poeciliopsis prolifica* [42]. All of these proteins are expressed in the human placenta, except for *Kiaa1324*, which is more active in the endometrium (Additional file 1: Table S1). In *P. prolifica*, we found expression of all of these genes in either placental or ovarian tissue, except for *Pla2g2a* (Additional file 1: Table S1).

As for the remaining nine positively selected genes in *H. formosa*, most are neuron associated (*Pnkd, Tmem230, Pcdh10, Gpr34, Slc35d3*) [43–47], which suggests ongoing selection on behavioural traits as observed earlier in poeciliids and teleost fish in general [48, 49]. The four remaining genes evolving under positive selection in *H. formosa* have varying or unknown functions. For a further elaboration on all genes found to be evolving under positive selection in *H. formosa*, see Additional file 1: Table S1.

To assess the function of positively selected genes in a quantitative manner, GO term enrichment analysis was performed using GOrilla [50]. The enriched GO terms with the lowest *p*-value were associated with cell-cell adhesion. Other enriched GO terms of interest were negative regulation of endopeptidase activity, dopamine and catecholamine metabolism, positive regulation of cytosolic calcium ion concentration, and cell migration. For all results of the GO enrichment analysis, see Additional file 2: Table S2.

**Fig. 2:**
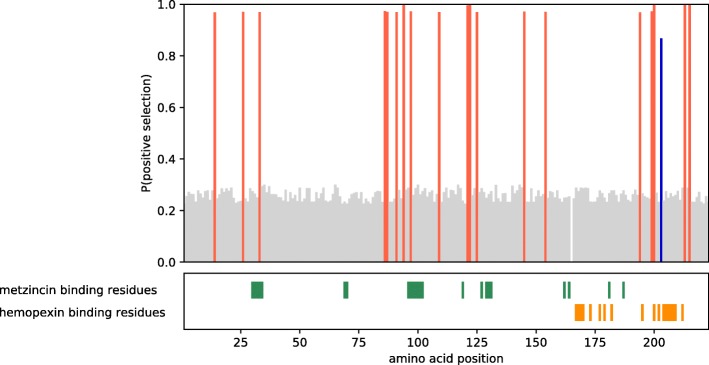
Likelihood of positive selection for each codon in *H. formosa* Timp4. Active residues are plotted on the bottom panel. Color codes for probability of positive selection: Red > 95% > blue > 80% > grey

**Fig. 3:**
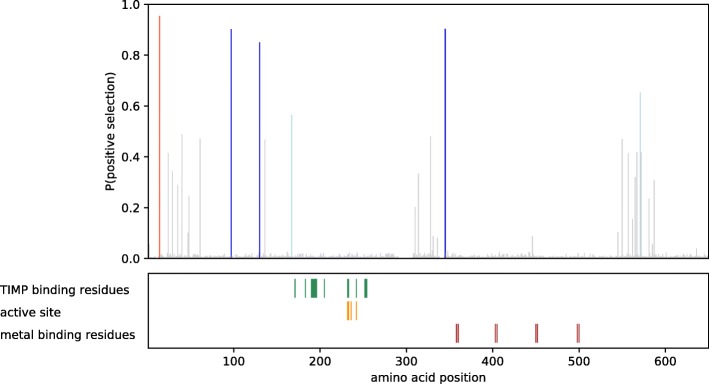
Likelihood of positive selection for each codon in *H. formosa* Mmp15. Active residues are plotted on the bottom panel. Color codes for probability of positive selection: Red > 95% > blue > 80% > light blue > 50% > grey

The evolution of complex structures may also involve changes in gene function and to investigate this possibility in *H. formosa*, we employed Bayes Empirical Bayes (BEB) analysis with PAML to infer which codons in the coding sequence are most likely subject to positive selection and thereby obtain information about a possible change of function. Two examples of this inference are shown for the *Timp4* and *Mmp15* genes (Figs. 2 and 3). As shown in the figure, positive selection in *H. formosa* Timp4 is widespread throughout the protein, as 20 out of 224 codons are predicted to be under positive selection (*p* > 80%). Positively selected sites interfere with residues of both the metzincin- as well as the hemopexin-binding domain, although most residues of these domains remain conserved. This may indicate a change in function, for instance in the type of metalloproteinases the protein binds to. Positively selected sites in Mmp15 are located next to and in between the catalytic and hemopexin (metal binding) domains, but do not overlap with the active residues. Little is known about these regions of the protein, but its catalytic function is not likely affected.

### Gene duplications

Potential gene duplications were identified by mapping reads from *H. formosa* to the *P. reticulata* genome, and identifying potential breakpoints by running Lumpy [51] on the alignment. Combining the breakpoints with a read depth signal allowed for identifying potential duplications. Using this method, we identified 46 potentially duplicated segments. However, after manual evaluation (see methods) only six of these segments were retained as likely true duplications, reflecting the difficulty to identify true duplications using short reads. These segments are given in Table 3.

**Table 3:** Duplicated regions in *H. formosa*

Duplicated area (position on *P. reticulata* genome)	Length (bp)	Genes
NC_024349.1:9797934-9803865	5931	Overlaps with *Cdh1*
NC_024331.1:4200605-4202283	1678	None
NC_024335.1:30069829-30071128	1299	Overlaps uncharacterized protein
NC_024338.1:15595450-15598894	3444	Contains *Pla2g2a*
NC_024345.1:3814448-3869038	54590	Overlaps with *Camk2g*, *Ccdc88a*
NC_024333.1:20591856-20595250	3394	Contains *Urah*

Although not many genes were found to be duplicated in *H. formosa*, the results are concordant with the results from the positive selection analysis. Firstly, the gene coding for 5-hydroxyisourate hydrolase (*Urah*) was duplicated. This gene belongs to the same uric acid degradation pathway as the positively selected gene allantoicase (*Allc*). Secondly, *Pla2g2a*, which we showed above to be evolving under positive selection, is duplicated completely. Thirdly, a cadherin protein (*Cdh1*) appeared partially duplicated, in addition to *Pcdh10* evolving under positive selection. *Cdh1* expression is also known to be regulated by *Btbd7* [52], a gene found to be positively selected in *H. formosa*. Finally, a relatively large duplication containing the majority of the *Camk2g* gene and a small part of the *Ccdc88a* gene was observed. Both of these genes are involved in neural development [53].

## Discussion

In this study, we sequenced and assembled the genome of *H. formosa*, a matrotrophic poeciliid. We aimed to use this information to gain insight in the evolution of the placenta in *H. formosa*, by looking for signatures of natural selection in its genome. One difficulty in identifying genes responsible for placentation is that natural selection in poeciliids is not limited to matrotrophy associated genes. For instance, immunity-related genes are consistently fast evolving in most vertebrate species, as a consequence of an evolutionary “arms race” between host immunity and pathogens (for instance [54, 55]). Furthermore, it is known that courtship behaviour is selected for in the family Poeciliidae [48, 56], which implies that many genes associated with behaviour are likely under the influence of sexual selection. These and other ongoing processes will cause coinciding genomic signatures of selection when considering selection acting on matrotrophy associated genes. We selected against these coinciding signatures of selection by distinguishing between positive selection across all investigated poeciliids and positive selection only observed in *H. formosa*, assuming that genes which are positively selected in both matrotrophic and lecithotrophic poeciliids are not likely responsible for the differences in placentation between the two groups.

Using this strategy, we identified 18 genes evolving under positive selection exclusively in *H. formosa*. Additionally, we identified six duplicated segments affecting a small number of genes. Significantly, mammalian orthologs of a substantial number of these genes are known to be involved in placental function and development, although most of these genes have different functions as well. For instance: protocadherin-10 (*Pcdh10*) is positively selected in *H. formosa*, and expressed in the human placenta [45]. Cadherins are known to be important for placental cell-cell adhesion [36]. However, *Pcdh10* is also involved in certain parts of the brain associated with visual and olfactory function [57], thus selective pressure on this gene could also occur because of selection on behavioural traits. Distinguishing between significance in placenta functioning or other functions was further evaluated by comparing gene function to the morphological differences between the placenta of *H. formosa* and that of its lecithotrophic relatives.

The main morphological differences in the placenta between matrotrophic and lecithotrophic poeciliids are found in the follicular epithelium, which is thicker and more extensively folded in matrotrophic species [12]. For a number of genes found to be positively selected in *H. formosa* it is possible they play a role in this change in tissue structure, most notably *Mmp15* and *Timp4*. Matrix metalloproteinases and their inhibitors are responsible for tissue remodelling [58], and both *Mmp15* and *Timp4* are active in the mammalian placenta [31, 32]. Therefore, it is plausible that positive selection acting on these genes could result in a difference in placental morphology. Similarly, claudins are also involved in endometrial tissue remodelling, by activating matrix metalloproteinases [37, 38]. Two claudin genes found to be positively selected are *Cldnd* and *Cldn4*. Yet another protein family involved in tissue remodelling are the cadherins, as these cell-cell adhesion proteins are involved in transducing the mechanical tension that regulates tissue remodelling [59, 60]. We found one cadherin (*Pcdh10*) to be positively selected in *H. formosa*, and another cadherin (*Cdh1*) to be partially duplicated in *H. formosa*. Because the duplicated *Cdh1* is a modular protein, consisting of six similar cadherin domains, a partial duplication could result in a functional protein. Both of these cadherins are expressed in the mammalian placenta, with *Cdh1* being essential for placental development in mice [36, 45, 61]. Finally, *Btbd7* is involved in tissue remodelling as a key regulator of cleft formation in branching morphogenesis [39]. In mammals, branching morphogenesis is an important mechanism in placental development [62]. As for poeciliids, much less is known about the mechanisms that regulate placenta formation, although cleft-like structures can be observed inside the folds of the follicular epithelium and branched microvilli in extensive matrotrophs [12, 63]. GO terms associated with these genes were also significantly enriched in positively selected genes in *H. formosa*, most notably “cell-cell adhesion via plasma-membrane adhesion molecules”, and “negative regulation of endopeptidase activity” (SI 2).

Molecular pathways other than those involved in tissue remodelling will also have played a role in placental development. For instance, a thicker follicular epithelium may result from an increased proliferation of the epithelial cells in *H. formosa*. Two of the positively selected genes identified are involved in endometrial cell proliferation in humans, namely *Rbl1* and *Kiaa1324* [33, 34].

Previous studies have shown that matrotrophic species carry an increased number of vesicles in their placental epithelial cells that are involved in trafficking nutrients from mother to embryo [13]. We found one gene involved in the regulation of vesicle trafficking to be positively selected in *H. formosa*, *Tmem230*. The involvement of Tmem230 in vesicle trafficking, however, has so far only been assessed in the brain [64]. *Tmem230* is expressed in the human placenta [41], but there is no literature on the function of *Tmem230* in this tissue.

These results give us a first insight into the genes that may be involved in the evolution of the placenta in *H. formosa*. Future studies should focus on generating genomic information for more species from different matrotrophic lineages in the family Poeciliidae [4]. Since the statistical power to detect positive selection is directly related to the number of species from different independent evolutionary lineages, adding genome information of more matrotrophic species and their closely related lecithotrophic ‘sister-species’ is likely to allow the detection of more matrotrophy-associated genes under positive selection. For example, an earlier study detected positive selection on the poeciliid *Igf2* gene using the protein-coding sequence of 38 teleost species (including 26 poeciliids), of which eight are extensive matrotrophs [20]. In our study, positive selection was not shown for *Igf2* (*p* = 0.12). This result may be a consequence of a different role of *Igf2* in *H. formosa* compared to other placental taxa, as it was shown that variation in *Igf2* expression is not correlated with changes in offspring size in *H. formosa* [65]. However, this different result may also be because using less (matrotrophic) species in the comparison reduces statistical power. In any case, genomic information for additional species will likely reveal other genes subject to positive selection that may have gone undetected in the present study. Additionally, this could also yield new insights into whether placental evolution in the different independent matrotrophic lineages is the result of selection on related or even the same genes, which would be an example of parallel evolution.

Finally, the low number of true duplications found in *H. formosa* reflects the difficulty of identifying duplicated segments using short read data only. To increase the amount of gene duplications that can be found, a reference genome of a matrotrophic poeciliid using long read or scaffolding information would be highly beneficial. Nevertheless, we were able to identify 18 genes that are exclusively selected in a highly matrotrophic species. Of these genes a high proportion is important in mammalian placenta function, suggesting convergence in the genetic building blocks of placental development between distantly related vertebrate lineages.

## Conclusions

We found 18 genes that show evidence of positive selection exclusively for the branch leading to the matrotrophic species *Heterandria formosa*, and not in any of the three lecithotrophic species in the family Poeciliidae that were used for comparison. Additionally, five (partial) gene duplications were identified in *H. formosa*. A substantial portion of these genes is involved in endometrial tissue remodelling and endometrial cell proliferation, consistent with morphological changes in the placenta of *H. formosa*. Based on these results, we hypothesize that the differences in placental morphology between lecithotrophic and (extensively) matrotrophic poeciliids are at least partly due to positive selection on genes involved in tissue remodelling and endometrial cell proliferation.

## Methods

### Whole genome sequencing of *Heterandria formosa*

*H. formosa* individuals were caught from Wakulla Springs under state permit number 07040111, after which they were transported to Leiden, the Netherlands, where they were kept in population tanks. An F3-generation female was sacrificed using a lethal dose of ms-222. DNA was isolated from the liver using the DNeasy kit from Qiagen, according to the manufacturers’ protocol. 1000 ng of DNA was sheared to a 100–800 bp range using a Covaris S-series sonicator. Genomic fragments were fit with adapters using the Paired-End DNA Sample Preparation Kit PE-102-1002 (Illumina inc.) and size-selected for 500 bp. Concentration and size profiles were determined on a Bioanalyzer 2100 using a High Sensitivity DNA chip. Paired-end sequencing was performed on an Illumina HiSeq 2000 sequencing system (Illumina Inc.) using the HiSeq Paired-End Cluster Generation Kit (PE-401-1001) and HiSeq Sequencing kit (FC-401-1001), yielding ~40X coverage of paired-end sequencing data.

### *Heterandria formosa* genome assembly

A de novo assembly of the genome of *H. formosa* was made using SPAdes 3.10.0 [23], with default settings. To estimate the genome size and heterozygosity beforehand, we performed k-mer counting (k = 20) using the Jellyfish software [66]. Redundant contigs due to heterozygosity of the sample were removed using redundans v0.13c [24] using default settings, and this tool was also used to rescaffold the assembly using paired-read information. After finishing of the assembly, we recalculated heterozygosity by mapping back the reads to the assembly with BWA 0.7.15 [67], removing PCR duplicates using SAMtools 1.5 [68], realigning using GATK 4.0 [69], before variant calling using the SAMtools mpileup and bcftools call commands [68], using default settings.

### Coding sequence alignments

Published reference genomes of *Poecilia reticulata*, *Poecilia formosa* and *Xiphophorus maculatus* were downloaded from the NCBI ftp server. A scan for orthologs between these genomes was performed using ProteinOrtho 5.16 [26], with settings -p = blastn+ and –sim = 0.8. We chose to only select 1:1:1 orthologs, of which we found 8,056. In order to locate these genes in the genome of *H. formosa*, a 1:1 alignment of the *H. formosa* assembly to the *P. reticulata* genome was created using LAST 810 [25], meaning that every nucleotide from the *H. formosa* genome can align to no more than one nucleotide of the *P. reticulata* genome, and vice versa. For all selected orthologs, the *H. formosa* sequence was then retrieved via this alignment. Only genes for which the coding sequence was completely covered by the whole genome alignment were selected for further analysis, which was the case for 6,774 genes. For these genes, four-way codon alignments of the coding sequence were made using PRANK v.170427 [70].

### Detecting positive selection

To detect positive selection, the codeml program of the PAML [27] package was used. This program provides a number of methods to detect positive selection, based on the ratio of non-synonymous versus synonymous substitutions (d_n_/d_s_), in the context of a known phylogenetic framework. A phylogenetic tree of the four species was constructed based on a PRANK alignment of the mitochondrial cytochrome b gene. For a neutrally evolving sequence, no distinction between synonymous and non-synonymous mutations is expected, and the d_N_/d_S_ ratio would approach 1. Protein-coding genes however, are expected to be conserved, so purifying selection against non-synonymous mutations is expected (d_N_/d_S_ < < 1). Indeed, on average, protein-coding genes have a d_N_/d_S_ ratio far below 1. However, certain situations can favour synonymous changes in a protein, for instance when a protein acquires a new (sub)function. This phenomenon is called positive selection and can lead to elevated d_N_/d_S_ ratios at some sites in the sequence, or branches in the phylogeny. PAML provides a number of models to test for the hypothesis that a gene is evolving under positive selection. For all analyses, we deleted columns with gaps in the alignment prior to analysis by using the PAML “cleandata” function. Although this leads to somewhat conservative results, it reduces false positives due to alignment gaps.

For this study, we use two models. Firstly, we use the site model to detect genes, which contain sites subject to positive selection across the entire phylogeny. For this, we compare the fit of a model allowing d_N_/d_S_ > 1 at certain codons in the coding sequence (model = 0, NSsites = 2) to a model where d_N_/d_S_ is not allowed to go above 1 (model = 0, NSsites = 1). The assumption is that genes subject to positive selection across the whole phylogeny are not likely to be matrotrophy-associated, as three out of four investigated species are lecithotrophic. Secondly, we use the branch-site model to test for positive selection in the phylogenetic branch leading to *H. formosa*. Here, again, a model allowing d_N_/d_S_ > 1 was compared to a model in which this is not the case, with d_N_/d_S_ able to vary within both amino acid positions and phylogenetic branches (model = 2, NSsites = 2, fix_omega = 0 for the selection model, model = 2, NSsites = 2, fix_omega = 1 for the neutral model). We chose *H. formosa* as the foreground branch, testing positive selection for this phylogenetic branch only.

*P*-values were obtained by performing likelihood ratio tests using a chi-square distribution (df = 2 for the site model, df = 1 for the branch-site model, as suggested in the PAML manual). Correction for multiple testing was performed using the Benjamini-Hochberg procedure [71], with 10% False Discovery Rate (FDR). Genes displaying significant positive selection in the branch leading to *H. formosa* were only kept in the analysis if they did not display significant positive selection for the site model. As an extra check, remaining genes were also tested for positive selection in all other branches of the phylogeny, and excluded from further analysis when this was the case. For positively selected genes belonging to gene families, 1:1 orthology was validated by aligning the *H. formosa* sequence against different *P. reticulata* paralogs of the corresponding gene family, so a false assessment of positive selection due to alignment to paralogs could be ruled out. Expression of identified genes was examined by performing blastn searches against the published transcriptome of the closely related *P. prolifica* [19], searching only against the placental and ovarian transcripts (Additional file 1: Table S1).

### GO term enrichment analysis

GO terms enriched in genes subject to positive selection were detected using GOrilla [50]. GOrilla takes a ranked list of genes and looks for GO terms occurring densely at the top of this list. For this, genes were ranked based on their *p*-value from the branch-site test, using *H. formosa* as foreground branch. We chose this method because the amount of positively selected genes in *H. formosa* was too small to find any enriched GO terms using ‘classical’ enrichment analysis (e.g. enriched GO terms in a list of significant genes compared to a background list).

### Detecting gene duplications

*H. formosa* sequencing reads were mapped on the *P. reticulata* genome using BWA 0.7.15 [67]. Duplicate read removal and realignment was performed using GATK 4.0 [69]. Breakpoints in the genome indicating potential copy number variations (CNVs) were detected by running Lumpy 0.2.13 [51] on the resulting alignment file. Because of the phylogenetic distance between *H. formosa* and *P. reticulata*, regions in the genome containing no mapped reads due to sequence divergence could not be distinguished from regions deleted in *H. formosa*. As a result, deletions could not be reliably assessed. Therefore, we focused on duplicated segments. Potential duplications were validated by comparing the read depth in a potentially duplicated segment to the average read depth of the alignment file, followed by visual evaluation in JBrowse [72]. For the validation of a potential duplication, four criteria were used. First, the read depth signal had to be at least 2 times the average read depth of the genome. Second, the coverage inside the putative duplication had to be even, that is, no coverage spikes because of repetitive elements that increase the average read depth. Third, a clear breakpoint on both sides of the CNV with discordant reads had to be visible. Fourth, only duplications with a minimum length of 1 kb were considered. To generate a set of *H. formosa*–specific duplications, resequencing libraries of *P. reticulata*, *P. formosa* and swordtail (*Xiphophorus hellerii*) were downloaded from GenBank, and the same analysis was performed for these species. If a putative duplication found in *H. formosa* was also found in one of these species, it was excluded from further analysis. Expression of genes that overlap with a duplication was examined by performing blastn searches against the published transcriptome of the closely related *P. prolifica* [19], searching only against the placental and ovarian transcripts (Additional file 1: Table S1).

## Additional files

Additional file 1: **Table S1.** Literature study on all genes positively selected exclusively in *H. formosa. (XLSX 16 kb)*

Additional file 2: **Table S2.** GO terms significantly enriched in positively selected genes in *H. formosa*, using a list of all tested genes ranked by *p*-value. (XLSX 11 kb)

## Data Availability

The sequence reads generated and analysed during the current study are available in the European Nucleotide Archive, under accession number PRJEB28818.

## Abbreviations

BEB: Bayes Empirical Bayes
FDR: False Discovery Rate
MI: Matroptrophy Index
